# Supplementary material for: Unveiling hidden ferrimagnetism and giant magnetoelectricity in polar magnet Fe2Mo3O8
Source: Sci Rep. 2015 Jul 21;5:12268. doi: 10.1038/srep12268 (PMC4508583; doi:10.1038/srep12268)
Supplement: Supplementary Information [file srep12268-s1.pdf]

Supplementary Information for

Unveiling hidden ferrimagnetism and giant magnetoelectricity in polar magnet $Fe_2Mo_3O_8$

Yazhong Wang[1], Gheorghe L. Pascut[1], Bin Gao[1], Trevor A. Tyson[1,2], Kristjan Haule[1], Valery Kiryukhin[1], and Sang-Wook Cheong[1,*]

[1]Rutgers Center for Emergent Materials and Department of Physics and Astronomy, Rutgers University, Piscataway, New Jersey 08854, USA

[2]Department of Physics, New Jersey Institute of Technology, Newark, New Jersey 07102, USA

[*]Corresponding author, sangc@physics.rutgers.edu

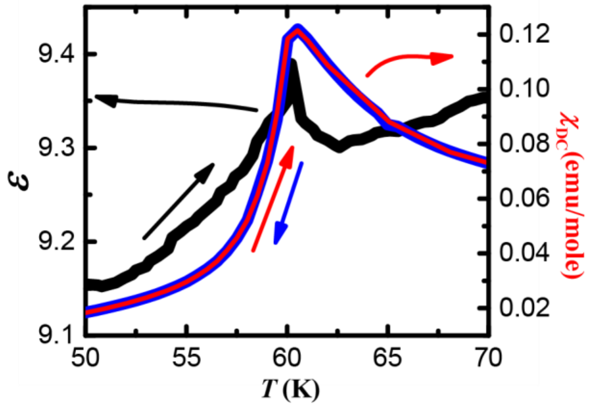

Supplementary Figure 1| Dielectric $\varepsilon(T)$ and magnetic $\chi_{//c}(T)$ susceptibilities in the vicinity of T_N. The magnetic susceptibility was measured on cooling in applied magnetic field H=0.2 T (blue line), and on subsequent warming in the same field (red line).

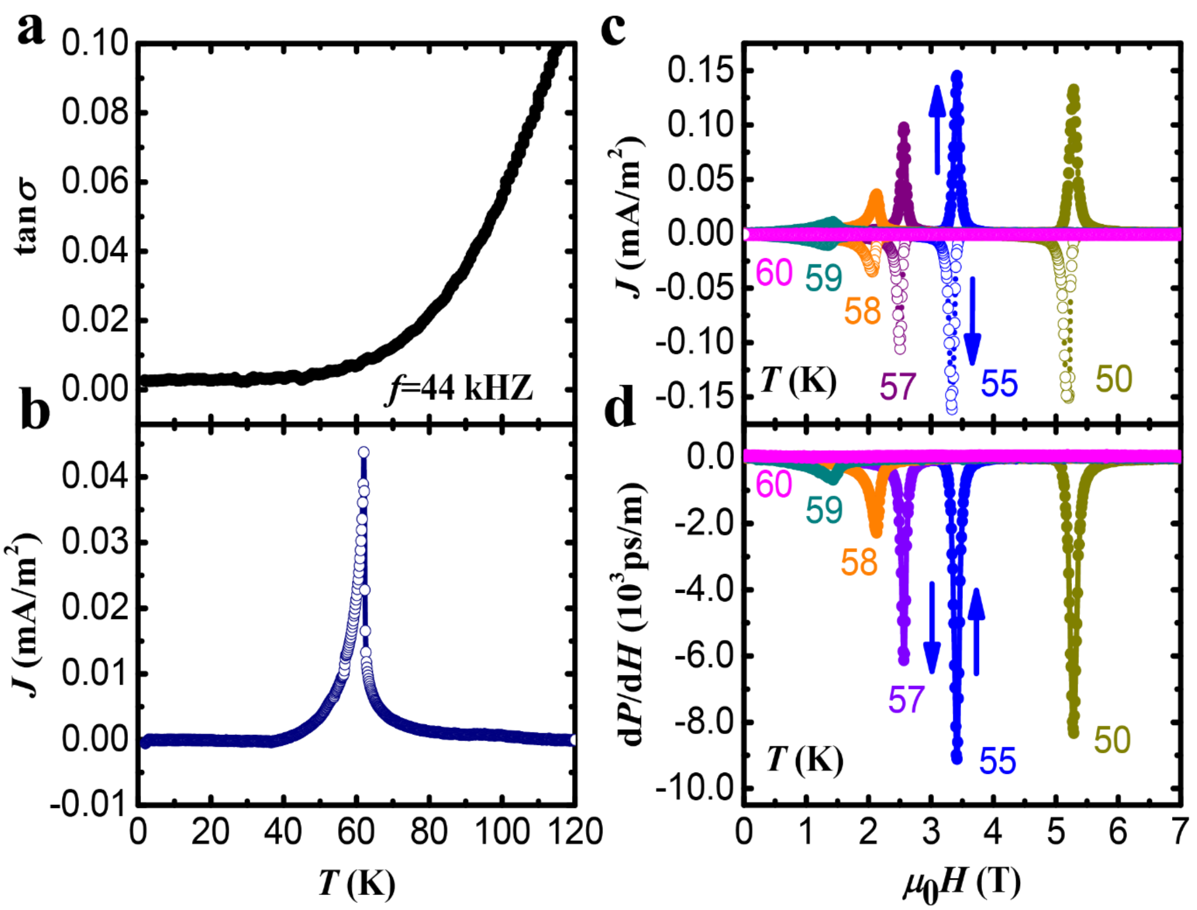

Supplementary Figure 2| Pyroelectric current, magneto-current, and the differential ME coefficient d*P*/d*H* at different temperatures. (a) Loss tangent as a function of temperature. **(b)** Pyroelectric current as a function of temperature, measured upon warming. **(c)** Isothermal magnetocurrent as a function of magnetic field. **(d)** Differential magnetoelectric coefficient d*P*/d*H* calculated using the data shown in panel (c). Arrows indicate the field sweeping direction. All the measurements are along the crystallographic *c* axis.

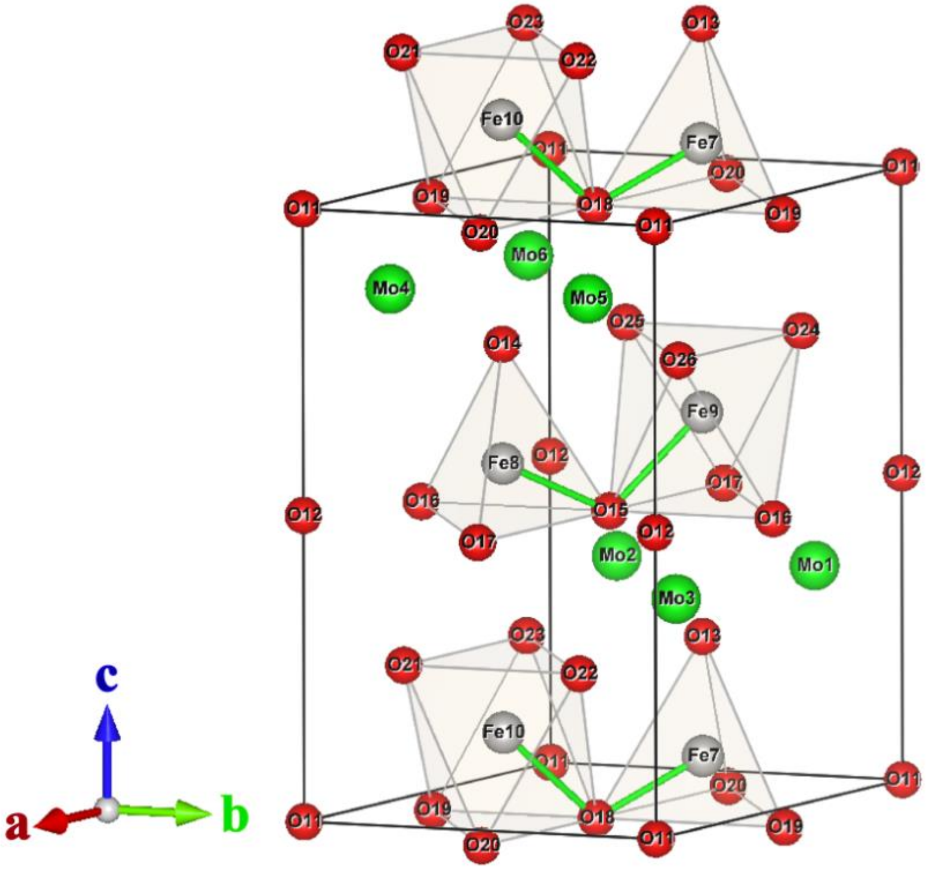

Supplementary Figure 3| Crystal structure of $Fe_2Mo_3O_8$, with ions labeled for Supplementary Table I below.

Ionic shifts in Å		
**Ion**	$10^3 \times \left(z_j^{AFM} - z_j^{PARA}\right) \times c$	$10^3 \times \left(z_j^{FRM} - z_j^{AFM}\right) \times c$
Mo1	2(2)	0(3)
Mo2	8(2)	-7(1)
Mo3	8(2)	-4(2)
Mo4	2(2)	0(3)
Mo5	8(2)	-7(1)
Mo6	8(2)	-4(2)
Fe7	10(2)	-5(2)
Fe8	10(2)	-5(2)
Fe9	5(2)	2(2)
Fe10	5(2)	2(2)
O11	10(2)	-3(2)
O12	10(2)	-3(2)
O13	11(2)	-3.5(1.0)
O14	11(2)	-3.5(1.0)
O15	47(10)	-30(12)
O16	-14(2)	30(4)
O17	-14(2)	17(3)
O18	47(10)	-30(12)
O19	-14(2)	30(4)
O20	-14(2)	17(3)
O21	-30(3)	15(10)
O22	6(2)	-9(2)
O23	6(2)	-15(8)
O24	-30(3)	15(10)
O25	6(2)	-9(2)
O26	6(2)	-15(8)

Supplementary Table I| Calculated ionic shifts for the paramagnetic to AFM transition (2nd column), and for the AFM to FRM transition (3rd column). See Supplementary Fig 3 for atomic labeling. Error bars reflect the impact of the multiple low-energy solutions characteristic to the GGA-PBE+U method.

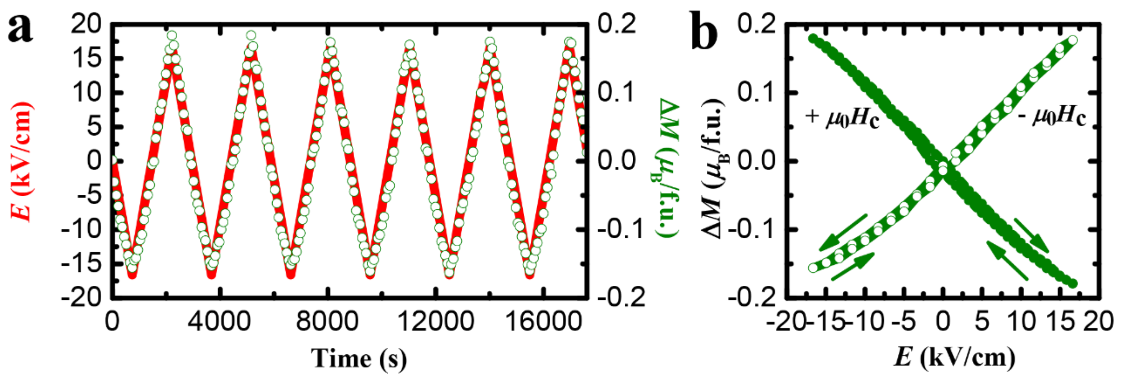

Supplementary Figure 4| Reversal of the differential magnetoelectric coefficient by changing the direction of the bias magnetic field. (a) Periodic modulation of magnetization (green) induced by an electric field (red) linearly varying between ±16.6 kV/cm, for *T*=55K and $\mu_0 H = -3.345$ T. These data differ from those shown in Fig 4(b) only by the direction of the applied magnetic field. Note the change of the sign of d*M*/d*E*. **(b)** Electric field dependence of magnetization for two opposite bias magnetic fields, +3.345 T, and -3.345 T. All the measurements are along the *c* axis.
